# Development of *Clostridium saccharoperbutylacetonicum* as a Whole Cell Biocatalyst for Production of Chirally Pure (*R*)-1,3-Butanediol

**DOI:** 10.3389/fbioe.2021.659895

**Published:** 2021-05-13

**Authors:** Alexander Grosse-Honebrink, Gareth T. Little, Zak Bean, Dana Heldt, Ruth H. M. Cornock, Klaus Winzer, Nigel P. Minton, Edward Green, Ying Zhang

**Affiliations:** ^1^Clostridia Research Group, BBSRC/EPSRC Synthetic Biology Research Centre (SBRC), Biodiscovery Institute, School of Life Sciences, University of Nottingham, Nottingham, United Kingdom; ^2^CHAIN Biotechnology Ltd., MediCity, Nottingham, United Kingdom

**Keywords:** *Clostridium saccharoperbutylacetonicum*, biotechnology, *phaB*, Allele Coupled Exchange, (*R*)-1, 3-butanediol, *Clostridium*

## Abstract

Chirally pure (*R*)-1,3-butanediol ((*R*)-1,3-BDO) is a valuable intermediate for the production of fragrances, pheromones, insecticides and antibiotics. Biotechnological production results in superior enantiomeric excess over chemical production and is therefore the preferred production route. In this study (*R*)-1,3-BDO was produced in the industrially important whole cell biocatalyst *Clostridium saccharoperbutylacetonicum* through expression of the enantio-specific *phaB* gene from *Cupriavidus necator*. The heterologous pathway was optimised in three ways: at the transcriptional level choosing strongly expressed promoters and comparing plasmid borne with chromosomal gene expression, at the translational level by optimising the codon usage of the gene to fit the inherent codon adaptation index of *C. saccharoperbutylacetonicum*, and at the enzyme level by introducing point mutations which led to increased enzymatic activity. The resulting whole cell catalyst produced up to 20 mM (1.8 g/l) (*R*)-1,3-BDO in non-optimised batch fermentation which is a promising starting position for economical production of this chiral chemical.

## Introduction

Chirally active compounds are much sought after by the chemical and pharmaceutical industry. The *R*-form of 1,3-butanediol (1,3-BDO), for example, is used for fragrances, pheromones, insecticides and as starting material for the production of azetidinone derivatives. The latter are intermediates of penem and carbapenem antibiotic synthesis (Nakatsuka et al., 1991; Matsuyama et al., 2001), a major area of development. Old β-lactam antibiotics lose their efficacy against bacteria due to emergent resistances which makes new discoveries crucial (Llarrull et al., 2010; Qin et al., 2014). This makes the cost-effective production of precursor molecules an important field of study.

Chemical production of (*R*)-1,3-BDO from threonine via asymmetric hydrogenation of 4-hydroxy-2-butanone (4H2B) has been studied, but satisfactory enantiomeric purity was not achieved (Larchevêque et al., 1991; Boaz et al., 2006). Enzymatic production of (*R*)-1,3-BDO has the potential to be cheaper than chemical production from petroleum-based substrates and its stereo-specificity is superior with up to 100% of the product yield having the desired conformation. Different whole cell catalysis approaches have been adopted with mixed results. Reduction through yeast fermentation of 4H2B led to low product yields when the desired stereo-specificity was reached (Matsuyama et al., 2001). However, later research showed promising results when reducing 4H2B using either newly isolated yeast strains, with up to 100% enantiomeric excess and a titre of 38.2 g/l (Zheng et al., 2012; Yang et al., 2014), or genetically engineered *E. coli*, where a 99% enantiomeric excess and 99% substrate yield were obtained (Itoh et al., 2007). Another approach using genetically modified *E. coli* based on the enantio-selective oxidation of (*S*)-1,3-BDO was initially promising (Matsuyama et al., 2001) but proved difficult to scale-up (Yamamoto et al., 2002).

As all of the above studies were reliant on relatively expensive substrates, the use of cheaper alternatives would benefit process economics. Two notable alternative routes to (*R*)-1,3-BDO have been explored. Nemr et al. (2018) assembled an elaborate metabolic pathway in *E. coli* that mediated the condensation of two pyruvate-derived molecules of acetaldehyde to form (*R*)-3-hydroxybutyrate ((*R*)-3-HB) and further to (*R*)-1,3-BDO. (*R*)-1,3-BDO yield equivalent to 13% of the theoretical maximum from glucose was achieved. In another approach, Kataoka et al. (2013) designed a synthetic pathway in *E. coli* encoding the acetyl-CoA acetyltransferase (*phaA*) and the acetoacetyl-CoA reductase (*phaB*) derived from *Cupriavidus necator*, combined with the *Clostridium saccharoperbutylacetonicum-*derived butyraldehyde dehydrogenase (*bld*) to produce 100.4 mM (9.05 g/l) (*R*)-1,3-BDO with 98.5% enantiomeric excess in batch fermentation. Fed-batch operation subsequently increased the product titre to 174.8 mM (15.8 g/l) (Kataoka et al., 2014).

*C. saccharoperbutylacetonicum* DSM 14923 (Hongo et al., 1968) is a widely used industrial acetone-butanol-ethanol- (ABE-) fermenting organism which is renowned for its particularly high *n*-butanol yields (Shaheen et al., 2000; Herman et al., 2017) and industrially favourable low sporulation capability (Keis et al., 2001). It has been found to grow on a wide range of industrially relevant first- and second-generation feedstocks, such as glucose, cellobiose, xylose, arabinose, mannose, galactose, pentose, starch and molasses (Shaheen et al., 2000; Yoshida et al., 2012; Yao et al., 2017). These characteristics make *C. saccharoperbutylacetonicum* an optimal whole cell biocatalyst for industrial applications. Since *C. saccharoperbutylacetonicum* possesses the necessary alcohol dehydrogenases and an inherently strong acetyl-CoA acetyltransferase (*thl*), introduction of the enantioselective PhaB from *C. necator* should be sufficient to produce (*R*)-1,3-BDO in this organism, obviating the need for multiple engineering steps. Green et al. (2017) expressed a *phaB* gene codon optimised for generic *Clostridium* spp. in *C. saccharoperbutylacetonicum* and observed 0.25 mM of (*R*)-1,3-BDO (plasmid expression) and 6 mM (*R*)-1,3-BDO (chromosomal integration) with no production of (*S*)-1,3-BDO. This study improves on earlier work engineering *C. saccharoperbutylacetonicum* for production of chirally pure (*R*)-1,3-BDO, through optimisation of PhaB-mediated catalysis at transcriptional, translational, enzyme and population levels.

## Materials and Methods

### Bacterial Strains, Growth, and Maintenance Conditions

*E. coli* HB101 (NEB, United Kingdom) was used as a host strain for plasmid propagation. *E. coli* was grown at 30°C or 37°C in Luria-Bertani (LB) broth or on LB-agar with 500 μg/ml erythromycin or 100 μg/ml spectinomycin. Unless stated otherwise, all chemicals were purchased from Sigma Aldrich, United Kingdom. The parent wild type strain *C. saccharoperbutylacetonicum* N1-4 (HMT) DSM14923 (Hongo et al., 1968) was purchased from the German collection of microorganisms and cell cultures GMBH (DSMZ). All *C. saccharoperbutylacetonicum* strains were grown in an anaerobic cabinet (Don Whitley, Yorkshire, United Kingdom) or tent (Coy Lab Products, United States) with artificial atmosphere (80:10:10 volume% nitrogen: carbon dioxide: hydrogen) at 37°C on solid RCM agar or on solid CBM (O’Brien and Morris, 1971). Standard liquid cultures were grown unshaken in TYA (Tashiro et al., 2004) with, per litre in g: yeast extract, 2; tryptone, 6; ammonium acetate, 3; MgSO_4_.7H_2_O, 0.3; KH_2_PO_4_, 0.5; FeSO_4_.7H_2_O, 0.01. Growth and solvent production assays of *C. saccharoperbutylacetonicum* were performed in CGM, 2xYTG, CBM, P2 and Biebl medium, with the following compositions per litre in g: CGM was prepared as described by Hartmanis and Gatenbeck with some modification (Hartmanis and Gatenbeck, 1984) yeast extract, 5; KH_2_PO_4_, 0.75; K_2_HPO_4_, 0.75; MgSO_4_.7H_2_O, 0.4; MnSO_4_.H_2_O, 0.01; FeSO_4_.7H_2_O, 0.01; NaCl, 1; asparagine, 2; (NH_4_)_2_SO_4_, 2; glucose, 50; CaCO_3_, 20. 2xYTG contained: yeast ectract, 10; tryptone, 16; NaCl, 5. CBM similar to O’Brien and Morris (1971), MgSO_4_.7H_2_O, 0.2; MnSO_4_.H_2_O, 0.00758; FeSO_4_.7H_2_O, 0.01; *p*-aminobenzoic acid, 0.001; biotin, 2 × 10^–6^; thiamine HCl, 0.001; casein hydrolysate, 4; K_2_HPO_4_, 0.5; KH_2_PO_4_, 0.5 (O’Brien and Morris, 1971). P2 similar to Baer et al. (1987), K_2_HPO_4_, 0.5; KH_2_PO_4_, 0.5; ammonium acetate, 2.2; MgSO_4_.7H_2_O, 2; MnSO_4_.H_2_O, 0.1; NaCl, 0.1; FeSO_4_.7H_2_O, 0.1; *p*-aminobenzoic acid, 0.1; thiamine, 0.1; biotine, 0.01 (Baer et al., 1987). Biebl media composition similar to Biebl, 2001: KH_2_PO_4_, 0.5; K_2_HPO_4_, 0.5; (NH_4_)_2_SO_4_, 5; MgSO_4_.7H_2_O, 0.2; CaCl_2_.2H_2_O, 0.02; FeSO_4_.7H_2_O, 0.00914; biotin, 25 × 10^–6^; 2 ml trace element solution made up of, per litre in mg: MnCl_2_.4H_2_O, 100; ZnCl_2_, 70; H_3_BO_3_, 60; NaMoO_4_.2H_2_O, 40; CoCl_2_.6H_2_O, 28.7; CuCl_2_.2H_2_O, 20; NiCl_2_.6H_2_O, 20; 1 ml 25% HCl (Biebl, 2001). All media were prepared with 50 g/l glucose and 20 g/l CaCO_3_ and adjusted to pH 6.2. For antibiotic selection in *C. saccharoperbutylacetonicum*, cultures were supplemented with 50 μg/ml erythromycin or 250 μg/ml spectinomycin.

Growth curves were done by growing *C. saccharoperbutylacetonicum* strains until mid-exponential phase for inoculation of triplicate 35 ml CGM broths to an OD_600_ of 0.05 in 250 ml serum bottles. The bottles were capped with rubber-butyl stoppers inside a cabinet and transferred outside the cabinet for crimping and static incubation at 37°C. Glucose, acids and solvents including (*R*)-3-HB and (*R*)-1,3-BDO were quantified with HPLC as previously described (Schwarz et al., 2017). Data was analysed with Graphpad PRISM 8.1 (Graphpad Software Inc., United States). The chirality of 3-HB and 1,3-BDO is determined using LC/MS analysis following DATAN (Diacetyl-L-Tartaric Anhydride) derivatisation (Struys et al., 2004).

### *Clostridium saccharoperbutylacetonicum* Electroporation

Electro-competent *Clostridium* cells were prepared based on the method described by Little et al. (2018). Culture in exponential phase was used to inoculate 300 ml TYA to OD_600_ 0.05 and grown to mid-exponential phase (OD_600_ 0.4–0.6). Cells were centrifuged at 7,500 rpm for 10 min and washed twice with ice-cold, reduced EPB buffer (270 mM Sucrose, 5 mM Na_2_HPO_4_, 5 mM NaH_2_PO_4_, pH 7.4), resuspended in 9 ml ice-cold EPB buffer containing 10% dimethyl sulphoxide and aliquots frozen at −80°C. For electroporation 580 μl of competent cells and typically 6 μg plasmid were added to a 4 mm electroporation cuvette inside an anaerobic cabinet. The mixture was kept on ice for 5 min before being electroporated with pulse settings of 2 kV, 25 μF and ∞Ω. Cells were immediately mixed with 10 ml warm TYA to recover for 6 h. Finally, cells were plated out on appropriate medium with appropriate antibiotics for selection.

### Plasmid Stability Assay

Plasmid segregational stability was determined as described previously (Heap et al., 2009). Briefly, *C. saccharoperbutylacetonicum* transformants were cultured for 12 h in TYA medium supplemented with the antibiotic erythromycin to select for the plasmid. The culture was washed twice in PBS to remove the antibiotic and then used to inoculate fresh, unsupplemented TYA medium at 1% (vol/vol). This marked the start (i.e., 0 h) of the stability experiment. The unsupplemented culture was then diluted 1% (vol/vol) into fresh medium every 12 h. At 12, 24, 36, 48, 60, 72, 84, and 96 h the culture was plated on both selective and non-selective agar to enumerate erythromycin-resistant colony-forming units and total colony-forming units.

### Plasmid Cloning

All restriction enzymes used were fast digest enzymes (ThermoFisher Scientific, United Kingdom), the ligase used throughout this work was the Rapid DNA Ligation Kit (ThermoFisher Scientific, United Kingdom). Plasmids were transformed into chemically competent *E. coli* HB101 according to supplier’s instructions (New England Biolabs, United Kingdom). Plasmids are listed in Supplementary Table 1 and primers and synthetic DNA fragments are listed in Supplementary Table 2.

### Plasmid pMTL83353::*phaB* and Derivatives

Plasmid pMTL83353::*phaB* (p_*phaB*) was cloned by digesting pMTL83251-p*fdx*_PhaB (Green et al., 2017) (featuring the *phaB* gene from *C. necator* N-1 codon optimised for *Clostridium spp*.) and pMTL83353 (Heap et al., 2009) with *Nde*I and *Nhe*I, ligating the fragment containing *phaB* into the pMTL83353 backbone. The point-mutated p_*phaB* derivatives were produced using the Quikchange site-directed mutagenesis kit according to supplier’s instruction (Agilent, United Kingdom) using primers Q47L_F and Q47L_R to produce pMTL83353::*phaB*_Q47L (p_*phaB*_L) and primers T173S_F and T173_R to produce pMTL83353::*phaB*_T173S (p_*phaB*_S). To produce the plasmid pMTL83353::*phaB*_Q47L_T173S (p_*phaB*_LS) carrying the *phaB* double-mutant, plasmid p_*phaB*_L was point-mutated using the Quikchange site directed mutagenesis kit as above with primers T173S_F and T173S_R.

The *C. necator phaB* gene was codon optimised to match the *C. saccharoperbutylacetonicum* codon adaptation index using the codon usage table calculated by Nakamura et al. at https://www.kazusa.or.jp/codon/(accessed 3/9/2019) (Nakamura et al., 2000). The codon optimised *phaB* fragment (*phaB*-opt) and the point mutated derivatives *phaB*-opt_Q47L, *phaB*-opt_T173S and *phaB*-opt_Q47L_T173S were ordered as synthetic DNA fragments (Twist Bioscience, United States) flanked by *Not*I and *Nde*I restriction sites upstream of the gene and *Hin*dIII and *Nhe*I downstream of the gene. The fragments were cloned into pMTL83353 (Heap et al., 2009) by digesting the plasmid and the fragments with *Nde*I and *Nhe*I and ligating each fragment separately into the digested backbone to obtain plasmids pMTL83353::*phaB*-opt_Q47L (p_*phaB*-opt_L), pMTL83353::*phaB*-opt_T173S (p_*phaB*-opt_S) and pMTL83353::*phaB*-opt_Q47L_T173S (p_*phaB*-opt_LS).

### ACE Plasmids

The Allele Coupled Exchange (ACE) (Heap et al., 2012) *pyrE* truncation plasmid pMTL-AGH21 (p_AGH21) was cloned by amplifying the following with Phusion High-Fidelity DNA Polymerase (New England Biolabs, United Kingdom): (i) a 300 bp fragment of the DSM14923 *pyrE* gene with primers pE_upstream_F and SOE_pE_trunc_up_R; (ii) the 282 bp multiple cloning site (MCS) from pMTL-ME6C (Heap et al., 2012)with primers SOE_pE_trunc_up_F and SOE_pE_trunc_dwn_R; (iii) the 1,200 bp downstream region of the DSM14923 *pyrE* locus with primers SOE_pE_trunc_dwn_F and pE_downstream_R. All three fragments were spliced together by splicing by overlap extension PCR (SOE-PCR) with primers pE_upstream_F and pE_downstream_R for ligation into pMTL85241 (Heap et al., 2009) at *Sbf*I and *Asc*I sites.

The cargo delivery ACE plasmid pMTL-AGH23 (p_AGH23) was cloned by amplifying the following: (i) a 626 bp fragment of *pyrE* with primers pE_upstream_F and SOE_pE_KI_up_R; (ii) a 323 bp fragment of the MCS from pMTL-ME6C (Heap et al., 2012) with primers SOE_pE_KI_up_F and SOE_pE_KI_dwn_R; (iii) a 1,200 bp fragment downstream of the DSM14923 *pyrE* locus with primers SOE_pE_KI_dwn_F and pE_downstream_R. The three fragments were used in a SOE-PCR as above with primers pE_upstream_F and pE_downstream_R, for ligation into pMTL85241.

Plasmid pMTL-AGH23::cspo_*fdx*-*phaB* was cloned by digesting p_*phaB* and p_AGH23 with enzymes *Not*I and *Nhe*I and ligating the resulting *phaB* fragment into the p_AGH23 backbone. The same process led to production of pMTL-AGH23::cspo_*fdx*-*phaB*-opt using plasmids p_AGH23 and p_*phaB*-opt and pMTL-AGH23::cspo_*fdx*-*phaB*-opt_Q47L_T173S using p_AGH23 and p_*phaB*-opt_LS. Plasmids pMTL-AGH23::cac_*ptb*-*phaB*-opt and pMTL-AGH23::cac_*ptb*-*phaB*-opt_Q47L_T173S were produced by exchanging the promoter P*_*cspo*_____*fdx*_* with promoter P*_*cac_*__*ptb*_* from plasmid pMTL8225::cac_*ptb*_*gusA* (p_*ptb*-*gusA*) through digestion of pMTL-AGH23::cspo_*fdx*-*phaB* or pMTL-AGH23::cac_*ptb*-*phaB*-opt and p_*ptb*-*gusA* with *Not*I and *Nde*I cloning the P*_*cac*_____*ptb*_* promoter into the 5,215 bp backbone.

### *gusA* Reporter Plasmids

Reporter plasmids to measure promoter strength were cloned fusing promoters from a promoter library (Willson, 2014) with a *gusA* (β-glucuronidase) gene. The latter was based on plasmid pMTL-JL1 (pMTL8514::fdx-*gusA*) which consisted of the *Streptococcus agalactiae gusA* gene (Wallace et al., 2015) under control of the *C. acetobutylicum* ferredoxin gene promoter in the modular shuttle vector pMTL85141. Plasmids pMTL8225::cac_araE-*gusA* (p_araE-*gusA*), pMTL8225::cac_adhE-*gusA* (p_adhE-*gusA*), pMTL8225::cac_*ptb*-*gusA* (p_*ptb*-*gusA*) and pMTL8225:: pj23119-*gusA* (p_pj-*gusA*) were constructed by digesting both pMTL8514::fdx-*gusA* and pMTL82251 (Heap et al., 2009) with *Not*I and *Nhe*I and ligating the fdx-*gusA* fragment into the pMTL82251 backbone. Plasmids pMTL8225::cbe_*fdx*-*gusA* (p_cbe_*fdx*-*gusA*), pMTL8225::cte_*fdx*-*gusA* (p_cte_*fdx*-*gusA*), pMTL8225::sac_*fdx*-*gusA* (p_sac_*fdx*-*gusA*), pMTL8225::clk_ *fdx*-*gusA* (p_clk_*fdx*-*gusA*), pMTL8225::cpf_*fdx*-*gusA* (p_cpf_*fdx*-*gusA*), pMTL8225::cbe_*thl*-*gusA* (p_cbe_*thl*-*gusA*), pMTL8225::ccv_*thl*-*gusA* (p_ccv_*thl*-*gusA*) and pMTL8225:: cby_*thl*-*gusA* (p_cby_thl-*gusA*) were cloned by digesting the corresponding pMTL82254 based plasmids (Willson, 2014; Supplementary Table 1) and p_*ptb*-*gusA* with enzymes *Not*I and *Nde*I and cloning the [promoter] fragment excised from pMTL82254::[promoter] into the backbone of p_*ptb*-*gusA*. The same process led to production of pMTL8225::cspo_*fdx*-*gusA* (p_cspo_*fdx*-*gusA*) by digestion of pMTL83353 (Heap et al., 2009) and p_*ptb*-*gusA* with *Not*I and *Nde*I and ligating the cspo_*fdx* fragment into the backbone.

### Allelic Exchange Mutant Generation

*C. saccharoperbutylacetonicum*Δ*pyrE* truncation and gene insertions downstream of *pyrE* were made as described by Heap et al. (2012). *C. saccharoperbutylacetonicum* DSM14923 was transformed with plasmid p_AGH21 followed by selection on RCM plates containing erythromycin. Faster growing colonies (potential single crossover integrants) were restreaked three times on CBM agar supplemented with 400 μg/ml 5-fluoroorotic acid and 20 μg/ml uracil and tested by colony PCR and subsequent Sanger sequencing (Eurofins Genomics, United Kingdom) for *pyrE* truncation and absence of plasmid. Colony PCR was performed as previously described for *Clostridium pasteurianum* (Pyne et al., 2013) with primers pE_genome_F and pE_genome_R. PCR products were sequenced with primer ACE_insert_seq_F. Plasmid loss was confirmed by colony PCR with primers 85XXX-RF and 8X2XX-RR.

Cargo integrated strains *C. saccharoperbutylacetonicum* Ω Cspo_*fdx*-*phaB* (Ω*fdx*-*phaB*), ΩCspo_*fdx*-*phaB*-opt (Ω*fdx*- *phaB*-opt), ΩCac_*ptb*-*phaB*-opt (Ω*ptb*-*phaB*-opt), ΩCspo_ *fdx*-*phaB*-opt_Q47L_T173S (Ω*fdx*-*phaB*-opt_LS) and ΩCac_*ptb*-*phaB*-opt_Q47L_T173S (Ω*ptb*-*phaB*-opt_LS) were produced by transforming the corresponding pMTL-AGH23 cargo plasmid into *C. saccharoperbutylacetonicum*Δ*pyrE* followed by direct selection for double-crossover integration on CBM plates without uracil. Integration was tested by PCR with primers pE_genome_F and pE_genome_R leading to a 2,836 bp fragment for Ω*fdx*-*phaB*, a 2,892 bp fragment for both P*_*cspo_*__*fdx*_* promoter based gene insertions and a 2,895 bp fragment for both P*_*cac_*__*ptb*_* promoter-based insertions if integration was successful, compared to 2,213 bp in *C. saccharoperbutylacetonicum*Δ*pyrE*. PCR products were sequenced with primers ACE_insert_seq_F and ACE_insert_seq_R. Plasmid loss was confirmed by PCR with primers 85XXX-RF and 8X2XX-RR.

### GusA Reporter Assay

Promoter strength in *C. saccharoperbutylacetonicum* was tested by measuring GusA activity when *gusA* was expressed from different promoters according to protocols by Miller (1972). Cell pellets from 1 ml cultures were defrosted and resuspended in up to 500 μl of Z-buffer (60 mM Na_2_HPO_4_, 45 mM NaH_2_PO_4_, 10 mM KCl, 1 mM MgSO_2_.7H_2_O, 50 mM β-mercaptoethanol, pH 8.5) to normalise OD_600_ across all samples. A 75 μl volume of the sample was transferred to a Corning 96 Well Black Polystyrene Microplate with clear bottom (Merck, United Kingdom) and OD_600_ measured in a Clariostar Plus plate reader (BMG Labtech, United Kingdom). Reaction was started by addition of 25 μl of 40 μg/ml 4-methylumbelliferyl-β-D-glucuronide dissolved in Z-buffer to each sample. Fluorescence was measured for 30 min at 450 nm after excitation at 360 nm at a distance of 7 mm and with a gain of 750. The resulting data was analysed in Excel (Microsoft Corporation, United States) by measuring slope of the regression line in the linear phase and the data was further analysed in GraphPad PRISM 8.1 (GraphPad Software Inc., United States).

## Results

**FIGURE 1:**
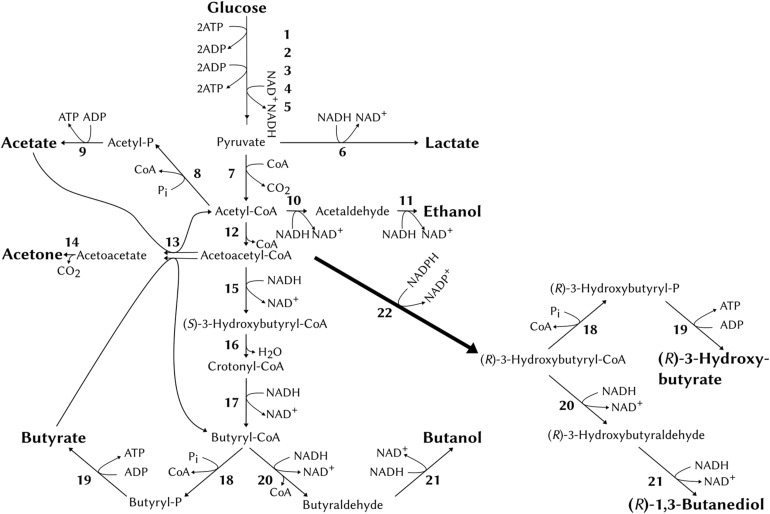
The natural acid- and solvent- producing pathways of *C. saccharoperbutylacetonicum* and the heterologous pathway from acetoacetyl-CoA via PhaB (acetoacetyl-CoA reductase, 22). Numbers correspond to enzymes as follows: 1, hexokinase; 2, phosphoglucose isomerase; 3, phosphofructokinase; 4, glyceraldehyde-3-phosphate dehydrogenase; 5, pyruvate kinase; 6, lactate dehydrogenase; 7, pyruvate-ferredoxin oxidoreductase; 8, phosphate acetyltransferase; 9, acetate kinase; 10, acetaldehyde dehydrogenase; 11, ethanol dehydrogenase; 12, thiolase; 13, acetoacetyl-CoA:acetate:CoA transferase/acetoacetyl-CoA:butyrate:CoA transferase; 14, acetoacetate decarboxylase; 15, β-hydroxybutyryl-CoA dehydrogenase; 16, crotonase; 17, butyryl-CoA dehydrogenase; 18, phosphotransbutyrylase; 19, butyrate kinase; 20, butyraldehyde dehydrogenase; 21, butanol dehydrogenase; 22, acetoacetyl-CoA reductase (*phaB*, from *Cupriavidus necator*).

Based on previous studies in *E. coli* (Kataoka et al., 2013), a pathway to chirally pure (*R*)-1,3-BDO in *C. saccharoperbutylacetonicum* was formulated based on the conversion of acetoacetyl-CoA to (*R*)-3-hydroxybutyryl-CoA using the *C. necator* enzyme acetoacetyl-CoA reductase encoded by *phaB*. In *E. coli* the condensation of two acetyl-CoA to one acetoacetyl-CoA was reliant on the over-expression of the *C. necator phaA* gene (coding for 3-ketothiolase). This was not necessary in *C. saccharoperbutylacetonicum* since the endogenous thiolase gene (*thlA*) is highly expressed (Wiesenborn et al., 1988). Furthermore, the endogenous enzymes alcohol- and aldehyde-alcohol dehydrogenases (*bdh* and *adh*) should catalyse the reactions from (*R*)-3-hydroxybutyryl-CoA via (*R*)-3-hydroxybutyraldehyde to (*R*)-1,3-BDO analogous to reduction of butyryl-CoA to butanol (Figure 1). The side product (*R*)-3-HB was expected to be produced predominantly through conversion of (*R*)-3-hydroxybutyryl-CoA via the endogenous phosphotransbutyrylase (*ptb*) to (*R*)-3-hydroxybutyryl-P and further via butyrate kinase (*buk*) to (*R*)-3-HB in analogy to the natural conversion of butyryl-CoA to butyrate. Additionally, some (*R*)-3-HB maybe produced by unspecific hydrolysis of the CoA thioester bond by endogenous thioesterases analogous to *E. coli* (Guevara-Martínez et al., 2019).

### Growth and Solvent Production

Various growth media were tested for solvent production from *C. saccharoperbutylacetonicum* DSM 14923 with the expectation that media supporting high solvent production would also promote (*R*)-1,3-BDO production (Supplementary Figure 1). The strain produced the highest yield of solvents per glucose (g solvents/g glucose) when grown in complex media (CGM and TYA) with 0.28 ± 0.02 g/g and 0.28 ± 0.00 g/g, respectively. In minimal Biebl medium the strain still produced 0.24 ± 0.00 g/g solvents. The highest solvent to acid ratio was found when growing on minimal media P2 or Biebl with 4.4 g/g (g solvents/g acids) and 4.3 g/g, respectively, while the ratio was 3.2 g/g for CGM and 3.5 g/g for TYA. However, since PhaB uses the four-carbon intermediate acetoacetyl-CoA to produce (*R*)-3-hydroxybutyryl-CoA, the 4-carbon to 2-carbon product ratio is more important for the production of (*R*)-1,3-BDO. This ratio was 5.1 g/g (g 4-carbon products/g 2-carbon products) for CGM, 3.5 g/g for TYA, 3.9 g/g for P2 and 5.0 g/g for Biebl medium. Thus, as it was expected that the highest yields of (*R*)-1,3-BDO yield in *C. saccharoperbutylacetonicum* strain would be obtained when grown in CGM, this medium was used throughout the rest of this work.

### Plasmid Stability Assays

**FIGURE 2:**
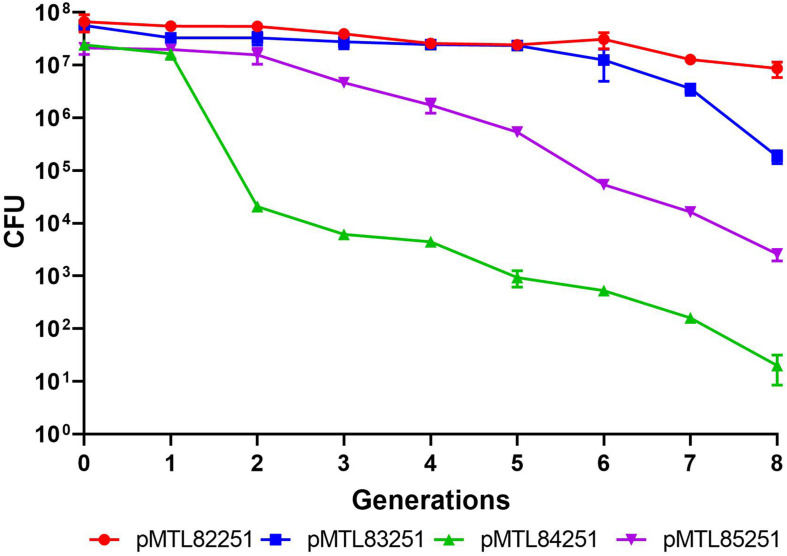
Plasmid stability assay testing four Gram-positive replicons in *C. saccharoperbutylacetonicum*. Plasmid pMTL82251 with pBP1 replicon in red circles, pMTL83251 with pCB102 replicon in blue squares, pMTL84251 with pCD6 replicon in green upward pointing triangles and pMTL85251 with pIM13 replicon in downward pointing triangles.

Four Gram-positive replicons (Heap et al., 2009) were tested for segregational stability in *C. saccharoperbutylacetonicum* by following the ratio of antibiotic resistant (plasmid containing) cells to total cell counts in non-selective media over time. The two most stable replicons were pBP1 and pCB102 over 8 generations which corresponded to approximately 96 h (Figure 2). Due to its reduced length of 1,625 bp compared to 2,403 bp of pBP1, the second most stable replicon pCB102 was chosen for plasmid-based expression of the heterologous *phaB* gene, as increasing plasmid size has been shown to negatively correlate with transformation efficiency (Hanahan, 1983).

### Plasmid Borne Expression of *phaB* With Increased Catalytic Activity

**FIGURE 3:**
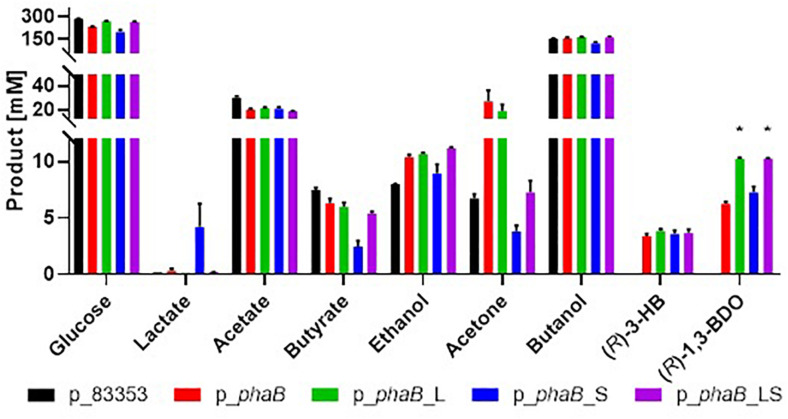
Comparison of product spectrum of *Cupriavidus necator* wild type *phaB* and the derivatives with point mutations and negative control without *phaB* (p_83353) expressed in *C. saccharoperbutylacetonicum* after 48 h. From **left** to **right** for each product p_83353 in black, p_*phaB* in red, p_*phaB*_L in green, p_*phaB*_S in blue, p_*phaB*_LS in purple. For glucose the consumed amount is shown for all other the produced amount. Error-bars represent SEM and * represents statistical significance against p_*phaB* (*t*-test, *p* < 0.05), *n* = 3.

Production of (*R*)-1,3-BDO was measured in *C. saccharoperbutylacetonicum* cells containing the plasmid p_*phaB* expressing the *C. necator phaB* (coding for acetoacetyl-CoA reductase) gene under the control of the promoter of the *Clostridium sporogenes fdx* gene (P*_*cspo_fdx*_*) (Figure 3, also Supplementary Figure 2). A control strain carrying the empty vector pMTL83353 was included for comparative purposes. Expression of *phaB* slowed growth of the strain considerably and the highest cell density was reached only after 48 h as compared to 24 h in the control. Strain p_*phaB* produced 6.3 ± 0.1 mM of (*R*)-1,3-BDO after 48 h while the control did not produce the compound, establishing that the pathway was functioning as intended. The by-product (*R*)-3-HB was produced at up to 5.1 ± 0.1 mM and is assumed to be reassimilated in the solventogenic phase via acetoacetate-CoA transferase (*ctfAB*) as it is the case for butyrate and acetate. The chemical chirality of 1,3-BDO and 3-HB were confirmed to be enantio-specific R form in previous study (Green et al., 2017). No S form of either compounds was found in the fermentation broth.

Point mutations Q47L and T173S in PhaB have previously been shown to increase activity and poly-3-HB accumulation in *Corynebacterium glutamicum* (Matsumoto et al., 2013) and in tobacco (Yokoo et al., 2015). Both point mutations were introduced separately into PhaB (plasmids p_*phaB*_L and p_*phaB*_S) and a double mutant (plasmid p_*phaB*_LS) was produced. *C. saccharoperbutylacetonicum* cells carrying plasmids p_*phaB*_L, p_*phaB*_S and p_*phaB*_LS produced 10.2 ± 0.2, 7.8 ± 0.1 and 10.3 ± 0.1 mM (*R*)-1,3-BDO, respectively (Figure 3).

### Codon Optimisation of Plasmid Expressed Enzyme

**FIGURE 4:**
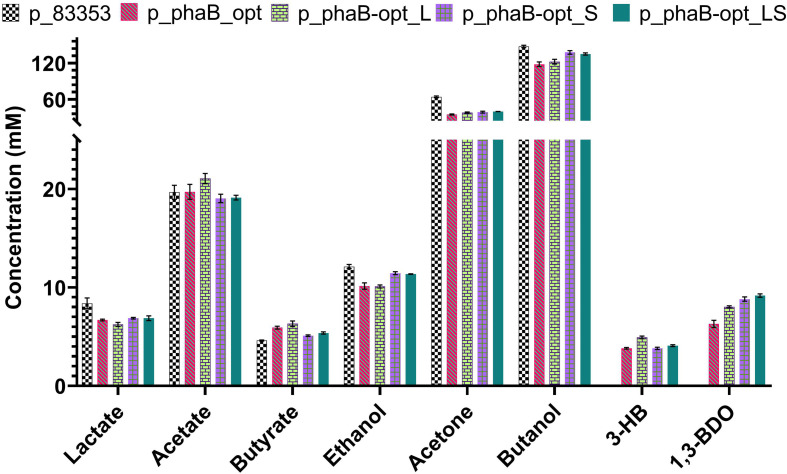
Comparison of product spectrum *C. saccharoperbutylacetonicum* cells carrying the empty plasmid control p_83353 and codon optimised *phaB* and its derivatives with point mutations after 48 h. From left to right for each product p_83353 in chequerboard, p_*phaB*_opt in red diagonal lines, p*_phaB*-opt_L in green bricks, p*_phaB*-opt_S in purple squares and p_phaB-opt_LS in green dots. Error-bars represent SEM.

The second option chosen to increase product titres after the point mutations, was to translationally optimise the heterologous *C. necator* gene through the alteration of its codon frequency to match that of *C. saccharoperbutylacetonicum*. Codon usage frequencies were determined using the database compiled by Nakamura and coworkers (Nakamura et al., 2000). Synonymous substitutions were made selecting codons with the highest frequency of usage in *C. saccharoperbutylacetonicum*. The resulting gene (*phaB*-opt), single point mutations (*phaB*-opt_L & *phaB*-opt_S) and the double point mutated gene (*phaB*-opt_LS) were ordered as synthetic constructs. Fermentation performance of the strains carrying codon optimised genes was tested against a plasmid control (Figure 4 and Supplementary Figure 3). Plasmids p_*phaB*-opt (6.3 ± 0.7 mM) and p_*phaB*-opt_S (8.8 ± 0.4 mM) produced slightly higher (*R*)-1,3-BDO concentrations while p_*phaB*-opt_L (8 ± 0.2 mM) and p_*phaB*-opt_LS (9.2 ± 0.3 mM) produced slightly less compared to the p_phaB based constructs.

### GusA Promoter Strength Assays

**FIGURE 5:**
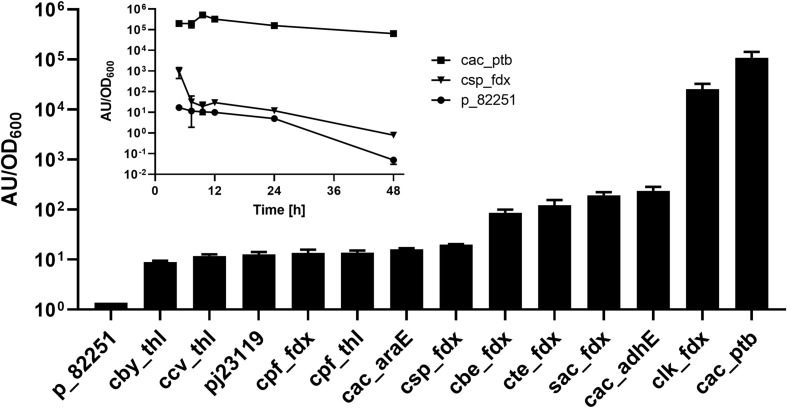
Promoter assay based on GusA production measured by accumulation of fluorescent product. A library of promoters derived from *fdx* and *thl* genes as well as a library of *C. acetobutylicum* promoters were tested for expression strength in *C. saccharoperbutylacetonicum*. Expression profiles over time of the strongest promoter P*_*cac_p*__*tb*_* and the standard promoter P*_*cspo_f*__*dx*_* were further assayed (inset graph). Error-bars represent SEM with *n* = 6 for all samples in the bar chart except p_82251 and P*_*cspo_f*__*dx*_* (*n* = 3) and *n* = 3 for all samples in the inset diagram.

Although several native *Clostridium* and synthetic promoters have been used for enzyme expression in *C. saccharoperbutylacetonicum* (Nakayama et al., 2008; Zhang et al., 2018) no systematic analysis of promoter strength has been published in this organism so far. A *gusA* (β-glucuronidase) reporter gene was employed to assess the relative strength of various clostridial promoters, including a library derived from the *fdx* and *thl* genes of several different clostridial species (Willson, 2014) as well as a synthetic promoter (iGEM part BBa_J23119). The *gusA* gene derived from *Streptococcus agalactiae* DSM 2134 was chosen because of its compatible GC content and codon usage as well as favourable kinetic properties of the encoded enzyme (Wallace et al., 2015), which were similar to those of *E. coli* GusA previously employed in *C. acetobutylicum* (Girbal et al., 2003). Relative promoter strength at 24 h post-inoculation was measured and the resulting *gusA* activities cluster into weak, mid-strength and strong promoters (Figure 5). Although the *C. sporogenes fdx* promoter (P*_*csp*__*o*___*fdx*_*) is known to be a strong promoter for expression of heterologous genes in *Clostridium* owing to its central position in the ABE-fermentation pathway (Minton et al., 2016), it was ineffective in *C. saccharoperbutylacetonicum*. The strongest promoter identified was that of the *C. acetobutylicum ptb* gene (P*_*cac*_____*ptb*_*) which was nearly 5400-fold stronger than P*_*csp*__*o*___*fdx*_*. Since the phosphotransbutyrylase (*ptb*) in *Clostridium* is only strongly expressed during exponential (acidogenic) phase in *C. acetobutylicum* (Girbal et al., 2003) and *Clostridium beijerinckii* (Ravagnani et al., 2000), GusA activity with expression from P*_*cac_ptb*_* and P*_*csp*__*o*___*fdx*_*promoters in *C. saccharoperbutylacetonicum* was measured over 48 h (Figure 5). The activity of P*_*cac_ptb*_* was high over the whole time-course, whilst *gusA* expression from the P*_*csp*__*o*___*fdx*_* promoter was only marginally higher than that of the empty vector control pMTL82251.

### *phaB* Chromosomally Integrated at the *pyrE* Locus

**FIGURE 6:**
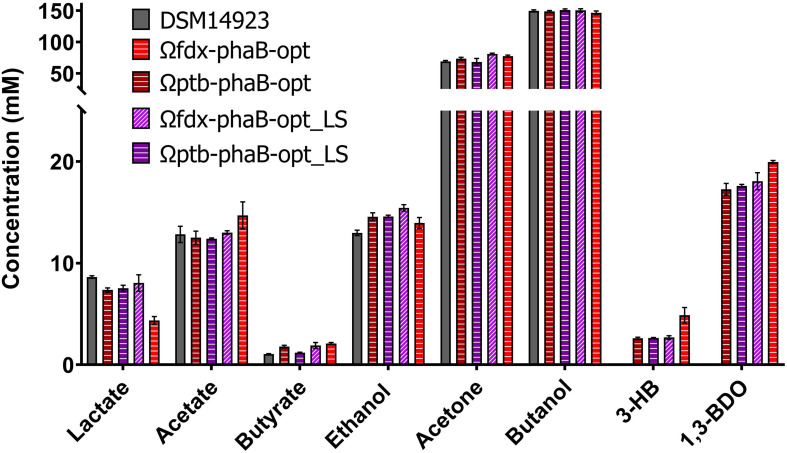
Comparison of product spectrum of the codon optimised *phaB* version and its derivatives encoding PhaB point mutations expressed in *C. saccharoperbutylacetonicum* by either weak promoter P*_*cspo_f*__*dx*_* or strong promoter P*_*cac_p*__*tb*_* from the chromosomal *pyrE* locus after 48 h. From left to right for each product DSM14923 in black, Ω*fdx*-*phaB*-opt in red horizontal lines, Ω*ptb*-*phaB*-opt in burgundy horizontal lines, Ω*fdx*-*phaB*-opt_LS in light purple diagonal lines and Ω*ptb*-*phaB*-opt_LS in purple horizontal lines. Error-bars represent SEM (*n* = 3).

The *C. necator phaB* gene was integrated into the *C. saccharoperbutylacetonicum* chromosome downstream of the *pyrE* gene using the ACE method (Heap et al., 2012). Selection for reversion to uracil prototrophy by restoration of the *pyrE* gene was used as a marker for successful cargo integration. A 3-fold higher (*R*)-1,3-BDO concentration of 19 ± 0.7 mM was produced after 48 h when *phaB* was expressed from the chromosome (Ωcspo_*fdx*-*phaB*) (Figure 6), than when expressed on a plasmid (p_*phaB*). A two-fold increase in product titre was obtained compared to the best performing plasmid-based codon optimised mutant (p_*phaB*-opt_LS).

Production of (*R*)-1,3-BDO by chromosomal expression of codon optimised *phaB* (*phaB-opt*) and the codon optimised double mutant (*phaB*-opt_LS) were measured from the differing strength P*_*cac_ptb*_* and P*_*csp*__*o*___*fdx*_* promoters. All the codon optimised genes produced increased (*R*)-1,3-BDO concentrations compared to the p_phaB sequence. The weak P*_*csp*__*o*___*fdx*_* promoter (Ω*fdx*-*phaB*-opt: 20 ± 0.1 mM; Ω*fdx*-*phaB*-opt_LS: 18 ± 1.6 mM) performed marginally better than the strong P*_*cac_ptb*_* promoter (Ω*ptb*-*phaB*-opt 17.3 ± 1.1 mM; Ω*ptb*-*phaB*-opt_LS 17.6 ± 0.3 mM) (Figure 6).

## Discussion

Different growth conditions were tested for maximal solvent production in *C. saccharoperbutylacetonicum* and a stable plasmid selected for expression of heterologous *phaB*. Having shown that expression of *phaB* leads to (*R*)-1,3-BDO production in *C. saccharoperbutylacetonicum*, options to increase the production titre were analysed. Three possibilities on different levels of protein expression were considered. Firstly, at an enzyme level, the catalytic activity of PhaB can be increased through point mutations in sites which were identified previously (Matsumoto et al., 2013). Secondly, translational efficiency can be increased by codon optimisation of the gene to match it to the natural codon adaptation of *C. saccharoperbutylacetonicum* (Quax et al., 2015). And lastly, increasing mRNA levels through boosting of transcription efficiency can be achieved through the use of strong promoters (Deuschle et al., 1986).

### Point Mutations in *phaB* Result in a Small Increase of *(R)*-1,3-BDO Production

Matsumoto et al. (2013) engineered and tested a library of approximately 20,000 *phaB* mutants generated by error prone PCR of which two variant genes were isolated that encoded an enzyme that exhibited increased *k*_*cat*_ values compared to the wild type enzyme. In one mutant a glutamine at position 47 was changed to leucine (Q47L) and in the other mutant a threonine at position 173 was changed to serine (T173S). Both mutated enzymes showed increased activity and poly-3-hydroxybutyrate accumulation when heterologously expressed in *Corynebacterium glutamicum* (Matsumoto et al., 2013) and in tobacco (Yokoo et al., 2015). It was hypothesised that introduction of these point mutations in *phaB* and expression in *C. saccharoperbutylacetonicum* should analogously lead to higher metabolic flux toward (*R*)-1,3-BDO. Both point mutations were introduced separately and a double mutant carrying both amino acid changes was created. Both single mutants exhibited the desired effect of increased (*R*)-1,3-BDO, however, the double mutant did not show an accumulative titre increase (Figure 3).

PhaB Q47L mutant (p_*phaB*_L) produced the highest amount of (*R*)-1,3-BDO, 1.6-fold higher than the PhaB wild type, while the T173S mutant (p_*phaB*_S) only showed a 1.2-fold increase of product titre. The double mutant produced the same amount as p_*phaB*_L which suggested either product inhibition at this level or the fact that the mutations cannot function in conjunction. Product inhibition can be ruled out by a growth assay in increasing *meso*-1,3-BDO concentrations which found *C. saccharoperbutylacetonicum* to grow uninhibited up to 300 mM of *meso*-1,3-BDO (Supplementary Figure 4). On the other hand, the fact that the effects of the two mutations would not accumulate could be explained based on data collected by Matsumoto et al. (2013). They found that PhaB T173S increases k*_*cat*_*/K*_*m*_*_(Acetoacetate–CoA)_ and decreases k*_*cat*_*/K*_*m*_*_(NADPH)_ while PhaB Q47L enhances k*_*cat*_*/K*_*m*_*_(NADPH)_ and decreases k*_*cat*_*/K*_*m*_*_(Acetoacetate–CoA)_. Therefore, the positive effects on catalytic enzyme activity of each mutation could cancel the effect of the other.

It was interesting to note that both p_*phaB* and p_*phaB*_S had a detrimental effect on *C. saccharoperbutylacetonicum* growth, evident by a slower growth rate and longer fermentation time (Supplementary Figure 2). Both strains also used less glucose than the control. This effect was reversed in p_*phaB*_L and p_*phaB*_LS. Suggesting that the Q47L mutation offsets the deleterious effect of *phaB* expression while increasing product formation. The deleterious effects of *phaB* on *C. saccharoperbutylacetonicum* growth were not observed in any of the codon optimised versions of the gene analysed (Supplementary Figures 3, 6). Since the only difference between the native *phaB* and *phaB*_L are two nucleotides (138-CAG and 130-CTG, respectively) and the same two nucleotides were altered to 138-CAA in *phaB*-opt (Supplementary Figure 5) it can be assumed that the deleterious effect stems from translational complications in this region. Correddu et al. (2019) found that co-occurrence of certain amino acid codons in *E. coli* led to a premature stop in the protein chain potentially through a frameshift. Similar effects could be at play here. The codon adaptation frequency pattern found in the vicinity of the mutation, however, does not suggest codon rarity to be a factor (Supplementary Figure 5), since the point mutated version uses an uncommon (in *C. saccharoperbutylacetonicum*) CTG codon for lysine (Nakamura et al., 2000). Thus, the ultimate cause of the observed deleterious effect cannot be ascertained.

### Codon Optimisation of *phaB* Has Minimal Impact on Product Concentration

Although codon optimisation was expected to lead to higher product yield, this was not found to be the case here (Figure 4). This was not entirely unexpected as codon optimisation based on the codon adaption index of an organism is relatively random with no significant correlation between the use of frequent codons and protein expression (Kudla et al., 2009). Future work should include codon harmonisation, where the relative native codon frequency per codon location found in *C. necator* is synchronised in the heterologous host organism. This approach takes into consideration mRNA regions presenting codons with low relative frequency (or codon adaptation index, CAI) which are hypothesised to slow down translation and allowing correct folding of the protein (Quax et al., 2015). Furthermore, it was shown that the 5’ mRNA leading sequence has a big influence on protein expression and surprisingly it was found that these sequences are preferably made up of rare codons, allowing appropriate ribosome distribution on the coding mRNA (Kudla et al., 2009; Quax et al., 2015).

### Chromosomal Integration Significantly Increases *(R)*-1,3-BDO Formation

**FIGURE 7:**
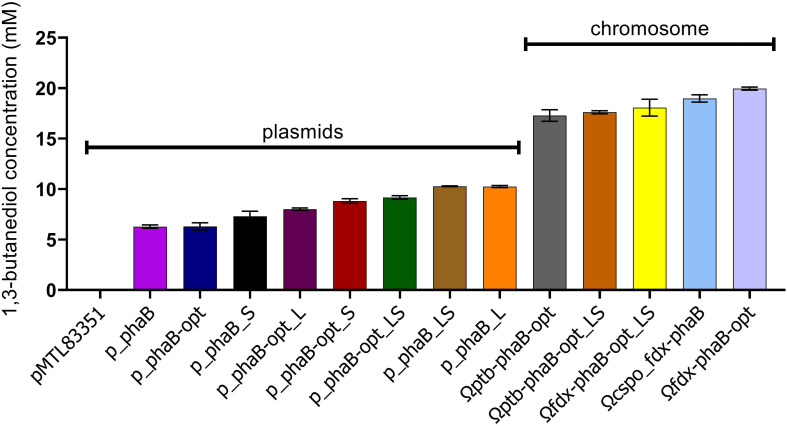
1,3-butanediol production from all the different constructs tested in this study. Error-bars represent SEM (*n* = 3).

Concomitant with testing the influence of codon optimisation on product titres the consequence of locating the heterologous *phaB* to the host chromosome as opposed to an autonomous plasmid was evaluated. Stable chromosomal integration is key to biotechnological exploitation of this technology by abolishing the need for antibiotics in fermentation and overcoming the inherent instability of replicative plasmids (Friehs, 2004; Heap et al., 2012). Expression was expected to be lower when the gene was expressed from the chromosome since there are more copies of the plasmid present than of the chromosome. This higher copy number would hypothetically translate into more mRNA and subsequently more protein, which in turn should be reflected in increased product titres. However, 3-fold higher (*R*)-1,3-BDO production was found in the mutant expressing *phaB* from the chromosome (Ωcspo_*fdx*-*phaB*) compared with it being expressed from a plasmid (p_*phaB*) and double the product titres compared to the best performing plasmid-based codon optimised mutant (p_*phaB*-opt_LS) were found (Figure 7). This effect was also previously reported by our team member (Green et al., 2017).

This finding suggests that overexpression of *phaB* has a negative impact on the final product titres. This may be due to pathway overloading, protein aggregation or polymerisation, liquid phase separation or general resource exhaustion of the cell (Bolognesi and Lehner, 2018). If any of these overexpression related issues play a role, then further overexpression would be pointless. To test this hypothesis *phaB* and the double mutant (*phaB*_LS) and codon optimised versions were expressed from the chromosome under the strong P*_*cac_*__*ptb*_* promoter and tested against expression from the weak P*_*csp*__*o*___*fdx*_* promoter (Ω*fdx*-*phaB*-opt). Product titres for (*R*)-1,3-BDO were lower when *phaB*-opt was strongly overexpressed with P*_*cac*___*ptb*_* (Ω*ptb*-*phaB*-opt) or when the potentially more active double mutant was used (Ω*fdx*-*phaB*-opt_LS and Ω*ptb*-*phaB*-opt_LS). However, one-way ANOVA revealed that the reduction was not significant [*F*(3,7) = 0.71, *p* = 0.57].

These findings suggest that the earlier hypothesis was correct and more active or higher expressed *phaB* does not lead to increased product titres. Since not only overexpression but also increasing enzyme activity by introduction of point mutations led to decreased product titre (Ω*fdx*-*phaB*-opt compared to Ω*fdx*-*phaB*-opt_LS), protein burden on the cell (Eguchi et al., 2018), protein underwrapping (Birchler and Veitia, 2012) or protein aggregation (Rosano and Ceccarelli, 2014) as a cause can potentially be excluded. Instead, evidence points toward reactant or product inhibition of the reaction. It is not clear through what mechanism this inhibition arises. However, the unfamiliar *R* stereo form of the substrates (*R*)-3-hydroxybutyryl-CoA and (*R*)-3-hydroxybutyraldehyde may not be reduced efficiently by native enzymes butyraldehyde dehydrogenase and butanol dehydrogenase, leading to intracellular build up that may cause feedback inhibition. Moreover, similar effects have been reported before and were attributed to metabolic imbalances caused by highly overexpressed genes and high enzyme activity of heterologously expressed genes (Atsumi et al., 2008; Kataoka et al., 2013).

### Final Remarks

It was shown that (*R*)-1,3-BDO can be produced up to 20 mM (1.8 g/l) in a non-optimised batch fermentation with *C. saccharoperbutylacetonicum*. This is the first time (*R*)-1,3-BDO production from glucose has been shown outside of *E. coli* in an anaerobic non-optimised batch fermentation. Titres reached are similar to studies using plasmid borne expression of pyruvate decarboxylase from *Zymomonas mobilis* and deoxyribose-5-phosphate aldolase from *Bacillus halodurans* (Nemr et al., 2018; Kim et al., 2020) but lower than in studies using plasmid borne expression of PhaA and PhaB from *R. eutropha* coupled with expression of Bld from *C. saccharoperbutylacetonicum* (Kataoka et al., 2013, 2014) summarised in Table 1. Higher titres can be reached using 4-hydroxy-2-butanone as substrate (Itoh et al., 2007; Zheng et al., 2012; Yang et al., 2014), however glucose is more widely available and more ecenomical than 4-hydroxy-2-butanone. Finally, future work should aim at optimising the fermentation and at upscaling of the production which is easier with an anaerobic organism such as the one used in this work.

**TABLE 1:** Summary of literature to date presenting microbial production of (*R*)-1,3-BDO from different substrates.

**Highest titre of (*R*)-1,3-BDO [g/l]**	**Organism**	**Substrate**	**Notes**	**References**
49.5	*E. coli* JM109	4-Hydroxy-2-butanone with 2-propanol as co-substrate	Plasmid borne overexpression of *Leifsonia* alcohol dehydrogenase	Itoh et al., 2007
38.7	*Candida krusei* ZJB-09162	4-Hydroxy-2-butanone with glucose co-substrate	Optimised preparative scale bioconversion process	Zheng et al., 2012
38.3	*Pichia jadinii* HBY61	4-Hydroxy-2-butanone with glucose co-substrate	Optimised preparative scale bioconversion process	Yang et al., 2014
15.6	*E. coli* MG1655 *lacI*q	Glucose	Strain from Kataoka et al. (2013)_ in optimised fed batch fermentation	Kataoka et al., 2014
9.05	*E. coli* MG1655 *lacI*q	Glucose	Plasmid borne overexpression of *phaA* and *phaB* from *Ralstonia eutropha NBRC 102504 and bld*from *C. saccharoperbutylacetonicum* ATCC 27012 in optimised batch fermentation	Kataoka et al., 2013
2.4	*E. coli* BL21	Glucose	Plasmid borne expression of pyruvate decarboxylase (*Zymomonas mobilis*) and the deoxyribose-5-phosphate aldolase (*Bacillus halodurans*) in two stage pH controlled bioreactor fermentation	Nemr et al., 2018
1.1	*E. coli* LMSE51C	Glucose	Aerobic pH controlled fed-batch fermentation of strain from Nemr et al. (2018) with BH1352 harbouring mutations F160Y/M173I	Kim et al., 2020

Here, three non-exclusive approaches to increase production of (*R*)-1,3-BDO were tested and combined. Firstly, point mutations were applied to increase enzyme activity. Secondly, the gene was codon optimised to fit the *C. saccharoperbutylacetonicum* codon adaptation index. Lastly, the point-mutated and codon-optimised versions of *phaB* were expressed from the chromosome with differing strength promoters. However, it has been shown that increasing transcription and protein activity did not lead to increased product titres. On the contrary these alterations led to decreased product formation by unknown mechanisms. Future work should aim at investigating these imbalances, debottlenecking the downstream pathway (e.g., by overexpression of endogenous genes of the pathway) and optimising the fermentation to increase product titres further.

## Data Availability Statement

The original contributions presented in the study are included in the article/Supplementary Material, further inquiries can be directed to the corresponding author.

## Author Contributions

AG-H carried out the laboratory work, data analysis, and drafted the manuscript. ZB and DH provided materials and participated in the laboratory work. EG and NM helped design the study and edited the manuscript. GL and RC coordinated the final part of the study and drafted the manuscript. The manuscript was written through contributions of all authors. YZ conceived the study, oversaw its design and coordination, helped with the data analysis, and revised the manuscript. All authors read and approved the final manuscript.

## Conflict of Interest

EG, DH, and ZB work for CHAIN Biotechnology Ltd. which has interest in commercialisation of the present microbial platform. CHAIN Biotechnology Ltd. has filed a patent application encompassing the initial work described in this article, International Application No. PCT/GB2017/050601. The remaining authors declare that the research was conducted in the absence of any commercial or financial relationships that could be construed as a potential conflict of interest.
