# Supplementary material for: Development of Clostridium saccharoperbutylacetonicum as a Whole Cell Biocatalyst for Production of Chirally Pure (R)-1,3-Butanediol
Source: Front Bioeng Biotechnol. 2021 May 13;9:659895. doi: 10.3389/fbioe.2021.659895 (PMC8155681; doi:10.3389/fbioe.2021.659895)
Supplement: Supplementary file 1 [file Data_Sheet_1.pdf]

# Development of *Clostridium saccharoperbutylacetonicum* as a whole cell biocatalyst for production of chirally pure (*R*)-1,3-butanediol

**Alexander Grosse-Honebrink[1], Gareth T. Little[1], Zak Bean[2], Dana Heldt[2], Ruth H. M. Cornock[1], Klaus Winzer[1], Nigel P. Minton[1], Edward Green[2] and Ying Zhang[1]***

[1]Clostridia Research Group, BBSRC/EPSRC Synthetic Biology Research Centre (SBRC), Biodiscovery Institute, School of Life Sciences, University of Nottingham, Nottingham, UK.

[2]CHAIN Biotechnology Ltd, MediCity, Nottingham, UK

*Correspondence: Corresponding Author Ying.Zhang@nottingham.ac.uk

## *Supplementary Material*

Table S1: Plasmids and strains used in this study.

| Name | Abbreviation | Properties | Source |
|---|---|---|---|
| **pMTL80000 modular vector series** | | | |
| pMTL82251 | p_82251 | *E. coli- Clostridium* shuttle vector (*pBP1*, *ermB*, ColE1, *traj*) | Heap *et al.*, 2009 |
| pMTL83251 | p_83251 | *E. coli- Clostridium* shuttle vector (*pCB102*, *ermB*, ColE1, *traj*) | Heap *et al.*, 2009 |
| pMTL84251 | p_84251 | *E. coli- Clostridium* shuttle vector (*pCD6*, *ermB*, ColE1, *traj*) | Heap *et al.*, 2009 |
| pMTL85251 | p_85251 | *E. coli- Clostridium* shuttle vector (*pIM13*, *ermB*, ColE1, *traj*) | Heap *et al.*, 2009 |
| pMTL83253 | p_83253 | *E. coli- Clostridium* shuttle vector (*pCB102*, *ermB*, ColE1, *traj*, $P_{cspo\_fdx}$, MCS, $T_{Cpa\ fdx}$) | Heap *et al.*, 2009 |

| | | | |
|---|---|---|---|
| pMTL83353 | P_83353 | *E. coli- Clostridium* shuttle vector (*pCB102*, *aad9*, ColE1, *traj*, $P_{cspo\_fdx}$, MCS, $T_{Cpa\ fdx}$) | |
| pMTL85241 | P_85241 | *E. coli- Clostridium* shuttle vector (*pIM13*, *ermB*, ColE1, *traj*, MCS, $T_{Cpa\ fdx}$) | |
| **PhaB Expression plasmids** | | | |
| pMTL83251-pfdx_PhaB | | *Cupriavidus necator phaB* gene replaces MCS | Green *et al*., 2017 |
| pMTL83353::*phaB* | p_*phaB* | *phaB* gene from pMTL83251-fdx_PhaB cloned into pMTL83353 | |
| pMTL83353::*phaB*_Q47L | p_*phaB*_L | Introduction of point-mutation in *phaB* to change aminoacid at location 47 from Q to L | |
| pMTL83353::*phaB*_T173S | p_*phaB*_S | Introduction of point-mutation in *phaB* to change aminoacid at location 173 from T to S | This study |
| pMTL83353::*phaB*_Q47L_ T173S | p_*phaB*_LS | Introduction of double point-mutation in *phaB* to change both aminoacids as above | |
| pMTL83353::*phaB*-opt | p_*phaB*-opt | *phaB* gene codon optimised for *C. saccharoperbutylacetonicum* | |
| pMTL83353::*phaB*-opt_Q47L_T173S | p_*phaB*-opt_LS | *phaB* gene codon optimised for *C. saccharoperbutylacetonicum* with double point-mutation as above | |
| **Chromosomal Integration Plasmids** | | | |
| pMTL-ME6C | | *Clostridium acetobutylicum* ACE integration plasmid | Heap *et al*. 2012 |
| pMTL-AGH21 | p_AGH21 | *C. saccharoperbutylacetonicum pyrE* truncation plasmid with pMTL85241 backbone | This study |

| | | | |
|---|---|---|---|
| pMTL-AGH23 | p_AGH23 | *C. saccharoperbutylacetonicum pyrE* repair and cargo delivery plasmid with pMTL85241 backbone | |
| pMTL-AGH23::cspo_fdx-*phaB* | | *C. saccharoperbutylacetonicum* delivery plasmid for *phaB* with *Clostridium sporogenes fdx* promoter | |
| pMTL-AGH23::cspo_fdx-*phaB*-opt | | *C. saccharoperbutylacetonicum* delivery plasmid for codon optimised *phaB* with *C. sporogenes fdx* promoter | |
| pMTL-AGH23::cac_ptb-*phaB*-opt | | *C. saccharoperbutylacetonicum* delivery plasmid for codon optimised *phaB* with *C.acetobutylicum ptb* promoter | |
| pMTL-AGH23::cspo_fdx-*phaB*-opt_Q47L_T173S | | *C. saccharoperbutylacetonicum* delivery plasmid for codon optimised *phaB* with double point mutations with *C. sporogenes fdx* promoter | |
| pMTL-AGH23::cac_ptb-*phaB*-opt_Q47L_T173S | | *C. saccharoperbutylacetonicum* delivery plasmid for codon optimised *phaB* with double point mutations with *C. acetobutylicum ptb* promoter | |
| **Promoter Library Plasmids** | | | |
| pMTL82254::cte_fdx | | Reporter vector with *Clostridium tetani fdx* promoter in front of CatP reporter | |
| pMTL82254::cbe_fdx | | Reporter vector with *Clostridium beijerinckii fdx* promoter in front of CatP reporter | |
| pMTL82254::sac_fdx | | Reporter vector with *C. saccharoper fdx* promoter in front of CatP reporter | |
| pMTL82254::clk_fdx | | Reporter vector with *Clostridium kluyveri fdx* promoter in front of CatP reporter | |
| pMTL82254::cpf_fdx | | Reporter vector with *Clostridium perfringens fdx* promoter in front of CatP reporter | |

| | | | |
|---|---|---|---|
| pMTL82254::cbe_thl | | Reporter vector with *C. beijerinckii thl* promoter in front of CatP reporter | |
| pMTL82254::ccv_thl | | Reporter vector with *Clostridium cellulovorans thl* promoter in front of CatP reporter | |
| pMTL82254::cby_thl | | Reporter vector with *Clostridium butyricum thl* promoter in front of CatP reporter | |
| **GusA reporter plasmids** | | | |
| pMTL8514::fdx_gusA | pMTL-JL1 | Reporter vector with *C. acetobutylicum fdx* promoter in front of GusA reporter | This study |
| pMTL8514::cac_araE-*gusA* | | Reporter vector with *C. acetobutylicum araE* promoter in front of GusA reporter | This study |
| pMTL8514::cac_adhE-*gusA* | | Reporter vector with *C. acetobutylicum adhE* promoter in front of GusA reporter | |
| pMTL8514::cac_ptb-*gusA* | | Reporter vector with *C. acetobutylicum ptb* promoter in front of GusA reporter | |
| pMTL8514::pj23119-*gusA* | | Reporter vector with constitutive consensus promoter iGEM part BBa_J23119 in front of GusA reporter | |
| pMTL8225::cspo_fdx-*gusA* | p_cspo_fdx-*gusA* | Reporter vector with *C. sporogenes fdx* promoter in front of GusA reporter | This study |
| pMTL8225::cbe_fdx-*gusA* | p_cbe_fdx-*gusA* | Reporter vector with *C. beijerinckii fdx* promoter in front of GusA reporter | |
| pMTL8225::cte_fdx-g*usA* | p_cte_fdx-*gusA* | Reporter vector with *C. tetani fdx* promoter in front of GusA reporter | |
| pMTL8225::sac_fdx-*gusA* | p_sac_fdx-*gusA* | Reporter vector with *C. saccharoper fdx* promoter in front of GusA reporter | |

| | | | |
|---|---|---|---|
| pMTL8225::clk_fdx-*gusA* | p_clk_fdx-*gusA* | Reporter vector with *C.kluyveri fdx* promoter in front of GusA reporter | |
| pMTL8225::cpf_fdx-*gusA* | p_cpf_fdx-*gusA* | Reporter vector with *C. perfringens fdx* promoter in front of GusA reporter | |
| pMTL8225::cbe_thl-*gusA* | p_cbe_thl-*gusA* | Reporter vector with *C. beijerinckii thl* promoter in front of GusA reporter | |
| pMTL8225::ccv_thl-*gusA* | p_ccv_thl-*gusA* | Reporter vector with *C. cellulovorans thl* promoter in front of GusA reporter | |
| pMTL8225::cby_thl-*gusA* | p_cby_thl-*gusA* | Reporter vector with *C. butyricum thl* promoter in front of GusA reporter | |
| pMTL8225::cac_araE-*gusA* | p_araE-*gusA* | Reporter vector with *C. acetobutylicum* araE promoter in front of GusA reporter | |
| pMTL8225::cac_adhE-*gusA* | p_adhE-*gusA* | Reporter vector with *C. acetobutylicum adhE* promoter in front of GusA reporter | |
| pMTL8225::cac_ptb-*gusA* | p_ptb-*gusA* | Reporter vector with *C. acetobutylicum ptb* promoter in front of GusA reporter | |
| pMTL8225::pj23119-*gusA* | p_pj-*gusA* | Reporter vector with constitutive consensus promoter iGEM part BBa_J23119 in front of GusA reporter | |
| **Strains** | | | |
| *C. saccharoperbutylacetonicum*Δ*pyrE* | | *C. saccharoperbutylacetonicum* with truncated *pyrE* | This study |
| *C. saccharoperbutylacetonicum*Ωcspo_fdx-*phaB* | | *C. saccharoperbutylacetonicum* with *phaB* with *C. sporogenes fdx* promoter downstream of repaired *pyrE* | |
| *C. saccharoperbutylacetonicum*Ωcspo_fdx-*phaB*-opt | Ωfdx-*phaB*-opt | *C. saccharoperbutylacetonicum* with codon optimised *phaB* with *C. sporogenes fdx* promoter | |

| | | | |
|---|---|---|---|
| *C. saccharoperbutylacetonicum*Ωcac_ptb-*phaB*-opt | Ωptb-*phaB*-opt | *C. saccharoperbutylacetonicum* with codon optimised *phaB* with *C. acetobutylicum ptb* promoter | |
| *C. saccharoperbutylacetonicum*Ωcspo_fdx-*phaB*-opt_Q47L_T173S | Ωfdx-*phaB*-opt_LS | *C. saccharoperbutylacetonicum* with codon optimised *phaB* with double point mutations with *C. sporogenes fdx* promoter | |
| *C. saccharoperbutylacetonicum*Ωcac_ptb-*phaB*-opt_Q47L_T173S | Ωptb-*phaB*-opt_LS | *C. saccharoperbutylacetonicum* with codon optimised *phaB* with double point mutations with with *C. acetobutylicum ptb* promoter | |

Table S2: Primers and synthetic DNA used in this study.

| Name | Sequence | Description | Flanking |
|---|---|---|---|
| pE_upstream_F | ATATTACCTGCAGGTTTAACTTTTGGAGATTTTGTTACTAAGAGTGG | SOE-PCR for ACE *pyrE* truncation and cargo delivery plasmid | 5'-SbfI |
| pE_downstream_R | ATGATTGCGCGCCTCTAATTTACTTTACTCAAATGGTATAAAATTCCATATAG | | 5'-AscI |
| SOE_pE_trunc_up_F | GGTGATACTGGAATCCTATTAGGAAGTAAATTGCGGCCGCTGTATCCATATG | SOE-PCR for ACE *pyrE* truncation plasmid | |
| SOE_pE_trunc_up_R | CATATGGATACAGCGGCCGCAATTTACTTCCTAATAGGATTCCAGTATCACC | | |
| SOE_pE_trunc_dwn_F | CTGAATGGCGAATGGCGCTAGCCAAGATAAGTTATTCCTATATAGGTTCTAATAAAAATTGAATCC | | |
| SOE_pE_trunc_dwn_R | GGATTCAATTTTTATTAGAACCTATATAGGAATAACTTATCTTGGCTAGCGCCATTCGCCATTCAG | | |
| SOE_pE_KI_up_F | GAATAGATGAGTATTATAAACAATATGGTGCTAAATAAGCGGCCGCTGTATCCATATG | SOE-PCR for ACE cargo delivery plasmid | |
| SOE_pE_KI_up_R | CATATGGATACAGCGGCCGCTTATTTAGCACCATATTGTTTATAATACTCATCTATTC | | |

| | | | |
|---|---|---|---|
| SOE_pE_KI_dwn_F | CATTTGCAGGCTTCTTATTTTTATGCTAGCCAAGATAAGTTATTCCTATATAGGTTCTAATAAAAATTGAATCC | | |
| SOE_pE_KI_dwn_R | GGATTCAATTTTTATTAGAACCTATATAGGAATAACTTATCTTGGCTAGCATAAAAATAAGAAGCCTGCAAATG | | |
| pE_genome_F | CTTGGTGGAATTTGCAGTGGA | Amplify whole *pyrE* locus | |
| pE_genome_R | AGGACTTTGGCTGCGGATAT | | |
| ACE_insert_seq_F | TTAGGGGTGACGACTGCAAT | Amplify/sequence the insertion region downstream *pyrE* | |
| ACE_insert_seq_R | CTTCCACTTAGAACCCATCC | | |
| 85XXX-LR | TTCCGACGCTTATTCGCTTC | Sequence ACE plasmids | |
| 8XX4X-RF | AGCGGAAGAGCGCCCAATAC | | |
| 85XXX-RF | TACTAATGAGAGGCGACGAC | Amplify region on pMTL85241 based plasmids between Gram+ replicon and ermB | |
| 8X2XX-RR | CTGGAACATCTGTGGTATGG | | |
| traj_F | GCTTGGCAAGGTCATGATG | Amplify and sequence promoters and reporters on GusA reporter plasmids and phaB expression plasmids | |
| pCB102_R1 | CTGTTATGCCTTTTGACTATC | | |
| Q47L_F | TCAAAACCTAAAGCTTTTTGCAGTTCAAGCCATCTTTCTCTTC | QuikChange primer for substitution on Q47L in *phaB* | |
| Q47L_R | GAAGAGAAAGATGGCTTGAACTGCAAAAAGCTTTAGGTTTTGA | | |
| T173S_F | ATTAACAGTAACTCCCTTTGATGCAACTTCCTGTGCTAATG | QuikChange primer for substitution on T173S in *phaB* | |
| T173S_R | CATTAGCACAGGAAGTTGCATCAAAGGGAGTTACTGTTAAT | | |

| | | | |
|---|---|---|---|
| phaB-opt | TacACTGCGGCCGCCATATGACACAAAGAATAG<br>CTTATGTAACTGGAGGCATGGGTGGGATTGGA<br>ACAGCTATATGTCAAAGACTTGCAAAAGATGGT<br>TTTAGAGTTGTAGCTGGATGTGGTCCAAATAGT<br>CCAAGAAGAGAAAGATGGTTAGAACAACAAAA<br>GGCATTAGGATTTGATTTTGTTGCTTCAGAAGG<br>CAATGTAGCAGATTGGGATTCTACTAAAACAGC<br>TTTTGATAAAGTTAAAGCAGAAGTAGGAGAGG<br>TTGATGTACTAATTAATAACGCTGGGATAACTA<br>GAGATGTTGTATTCAGAAAGATGACAAGAGCA<br>GATTGGGATGCTGTTATCGACACTAATCTTACA<br>TCATTATTTAATGTAACTAAACAAGTTATTGATG<br>GCATGGCAGATCGTGGATGGGGCAGAATAGTA<br>AATATTTCTAGTGTTAATGGTCAAAAGGGGCAA<br>TTTGGACAAACAAATTATTCAACTGCTAAAGCA<br>GGCCTTCATGGTTTTACAATGGCTTTAGCACAA<br>GAAGTAGCTACTAAAGGAGTTACAGTAAATAC<br>TGTTTCTCCAGGGTATATAGCAACAGATATGGT<br>AAAGGCTATTAGACAAGATGTTCTTGATAAAAT<br>AGTAGGTACTATCCCTGTTAAAAGACTAGGAG<br>AACCAGAAGAAATTGCAAGTATATGTGCTTGGT<br>TATCATCAGATGAGAGTGGTTTTTCAACTGGAG<br>CAGATTTTTCTCTTAATGGTGGATTACACATGG<br>GCTAGAAGCTTGCTAGCTACACT | Synthetic DNA fragment with codon optimised *phaB* gene | 5'-NotI<br>/<br>NdeI; 3'-HindIII<br>/<br>NheI |
| phaB-opt_Q47L | TacACTGCGGCCGCCATATGACACAAAGAATAG<br>CTTATGTAACTGGAGGCATGGGTGGGATTGGA<br>ACAGCTATATGTCAAAGACTTGCAAAAGATGGT<br>TTTAGAGTTGTAGCTGGATGTGGTCCAAATAGT<br>CCAAGAAGAGAAAGATGGTTAGAATTACAAAA<br>GGCATTAGGATTTGATTTTGTTGCTTCAGAAGG<br>CAATGTAGCAGATTGGGATTCTACTAAAACAGC<br>TTTTGATAAAGTTAAAGCAGAAGTAGGAGAGG<br>TTGATGTACTAATTAATAACGCTGGGATAACTA<br>GAGATGTTGTATTCAGAAAGATGACAAGAGCA<br>GATTGGGATGCTGTTATCGACACTAATCTTACA<br>TCATTATTTAATGTAACTAAACAAGTTATTGATG<br>GCATGGCAGATCGTGGATGGGGCAGAATAGTA<br>AATATTTCTAGTGTTAATGGTCAAAAGGGGCAA<br>TTTGGACAAACAAATTATTCAACTGCTAAAGCA<br>GGCCTTCATGGTTTTACAATGGCTTTAGCACAA<br>GAAGTAGCTACTAAAGGAGTTACAGTAAATAC<br>TGTTTCTCCAGGGTATATAGCAACAGATATGGT<br>AAAGGCTATTAGACAAGATGTTCTTGATAAAAT<br>AGTAGGTACTATCCCTGTTAAAAGACTAGGAG<br>AACCAGAAGAAATTGCAAGTATATGTGCTTGGT<br>TATCATCAGATGAGAGTGGTTTTTCAACTGGAG | Synthetic DNA fragment with codon optimised *phaB* gene with Q47L point mutation | 5'-NotI<br>/<br>NdeI; 3'-HindIII<br>/<br>NheI |

| | | | |
|---|---|---|---|
| | CAGATTTTTCTCTTAATGGTGGATTACACATGG<br>GCTAGAAGCTTGCTAGCTACACT | | |
| phaB-opt_T173S | TacACTGCGGCCGCCATATGACACAAAGAATAG<br>CTTATGTAACTGGAGGCATGGGTGGGATTGGA<br>ACAGCTATATGTCAAAGACTTGCAAAAGATGGT<br>TTTAGAGTTGTAGCTGGATGTGGTCCAAATAGT<br>CCAAGAAGAGAAAGATGGTTAGAACAACAAAA<br>GGCATTAGGATTTGATTTTGTTGCTTCAGAAGG<br>CAATGTAGCAGATTGGGATTCTACTAAAACAGC<br>TTTTGATAAAGTTAAAGCAGAAGTAGGAGAGG<br>TTGATGTACTAATTAATAACGCTGGGATAACTA<br>GAGATGTTGTATTCAGAAAGATGACAAGAGCA<br>GATTGGGATGCTGTTATCGACACTAATCTTACA<br>TCATTATTTAATGTAACTAAACAAGTTATTGATG<br>GCATGGCAGATCGTGGATGGGGCAGAATAGTA<br>AATATTTCTAGTGTTAATGGTCAAAAGGGGCAA<br>TTTGGACAAACAAATTATTCAACTGCTAAAGCA<br>GGCCTTCATGGTTTTACAATGGCTTTAGCACAA<br>GAAGTAGCTTCAAAAGGAGTTACAGTAAATAC<br>TGTTTCTCCAGGGTATATAGCAACAGATATGGT<br>AAAGGCTATTAGACAAGATGTTCTTGATAAAAT<br>AGTAGGTACTATCCCTGTTAAAAGACTAGGAG<br>AACCAGAAGAAATTGCAAGTATATGTGCTTGGT<br>TATCATCAGATGAGAGTGGTTTTTCAACTGGAG<br>CAGATTTTTCTCTTAATGGTGGATTACACATGG<br>GCTAGAAGCTTGCTAGCTACACT | Synthetic DNA fragment with codon optimised *phaB* gene with T173S point mutation | 5'-NotI<br>/<br>NdeI; 3'-HindIII<br>/<br>NheI |
| phaB-opt_Q47L_T173S | TacACTGCGGCCGCCATATGACACAAAGAATAG<br>CTTATGTAACTGGAGGCATGGGTGGGATTGGA<br>ACAGCTATATGTCAAAGACTTGCAAAAGATGGT<br>TTTAGAGTTGTAGCTGGATGTGGTCCAAATAGT<br>CCAAGAAGAGAAAGATGGTTAGAATCACAAAA<br>GGCATTAGGATTTGATTTTGTTGCTTCAGAAGG<br>CAATGTAGCAGATTGGGATTCTACTAAAACAGC<br>TTTTGATAAAGTTAAAGCAGAAGTAGGAGAGG<br>TTGATGTACTAATTAATAACGCTGGGATAACTA<br>GAGATGTTGTATTCAGAAAGATGACAAGAGCA<br>GATTGGGATGCTGTTATCGACACTAATCTTACA<br>TCATTATTTAATGTAACTAAACAAGTTATTGATG<br>GCATGGCAGATCGTGGATGGGGCAGAATAGTA<br>AATATTTCTAGTGTTAATGGTCAAAAGGGGCAA<br>TTTGGACAAACAAATTATTCAACTGCTAAAGCA<br>GGCCTTCATGGTTTTACAATGGCTTTAGCACAA<br>GAAGTAGCTTCAAAAGGAGTTACAGTAAATAC<br>TGTTTCTCCAGGGTATATAGCAACAGATATGGT<br>AAAGGCTATTAGACAAGATGTTCTTGATAAAAT<br>AGTAGGTACTATCCCTGTTAAAAGACTAGGAG<br>AACCAGAAGAAATTGCAAGTATATGTGCTTGGT | Synthetic DNA fragment with codon optimised *phaB* gene with Q47L and T173S point mutation | 5'-NotI<br>/<br>NdeI; 3'-HindIII<br>/<br>NheI |

| | | | |
|---|---|---|---|
| | TATCATCAGATGAGAGTGGTTTTTCAACTGGAG CAGATTTTTCTCTTAATGGTGGATTACACATGG GCTAGAAGCTTGCTAGCTACACT | | |

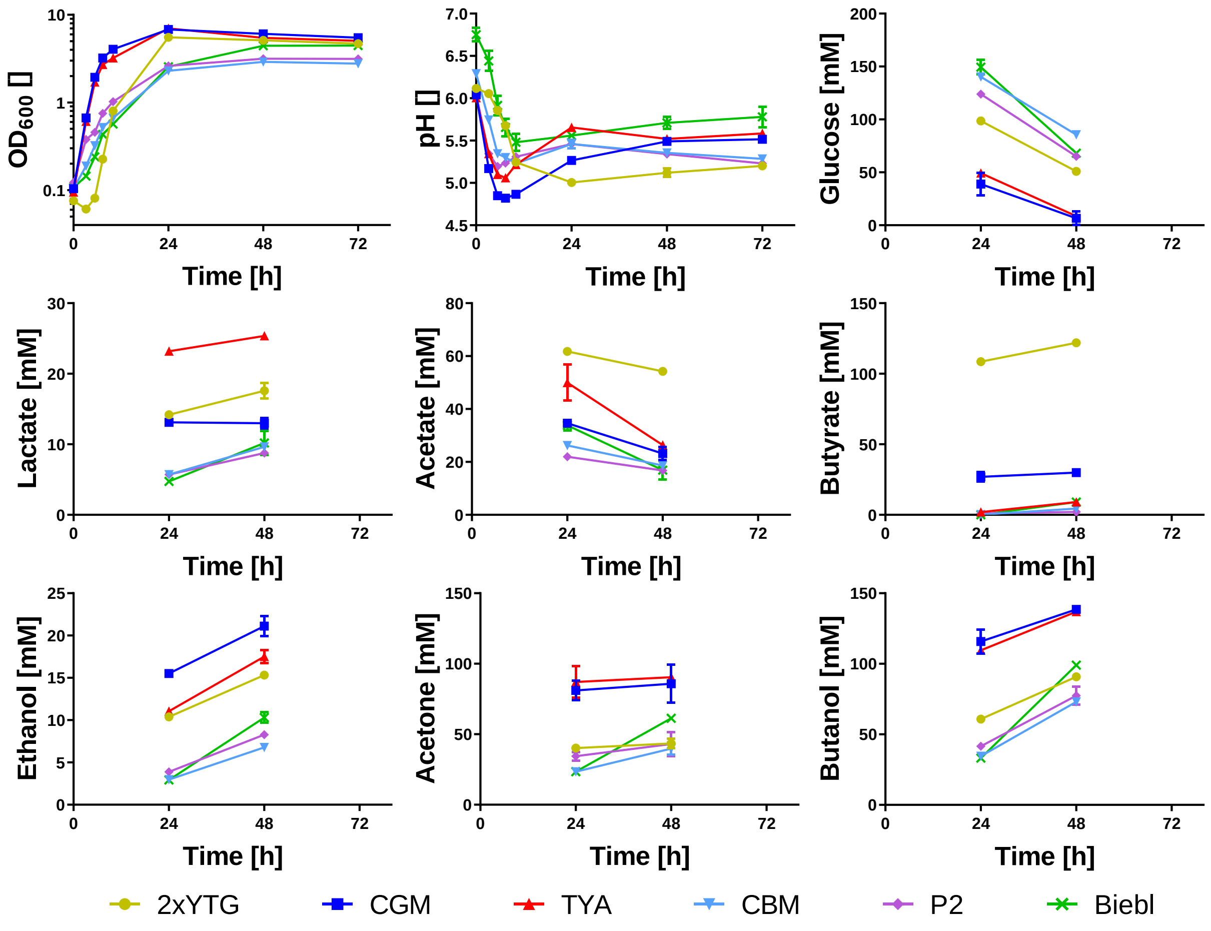


Figure S1. Different media were tested with *C. saccharoperbutylacetonicum* for best solvent production which should hypothetically lead to more (*R*)-1,3-BDO production in a strain with heterologous *phaB* expressed. *C. saccharoperbutylacetonicum* was grown in 2xYTG in yellow dots, CGM in blue squares, TYA in red upwards pointing triangles, CBM in pale blue downward pointing triangles, P2 medium in purple diamonds and Biebl medium in green x's. Error bars represent SEM, n= 2.

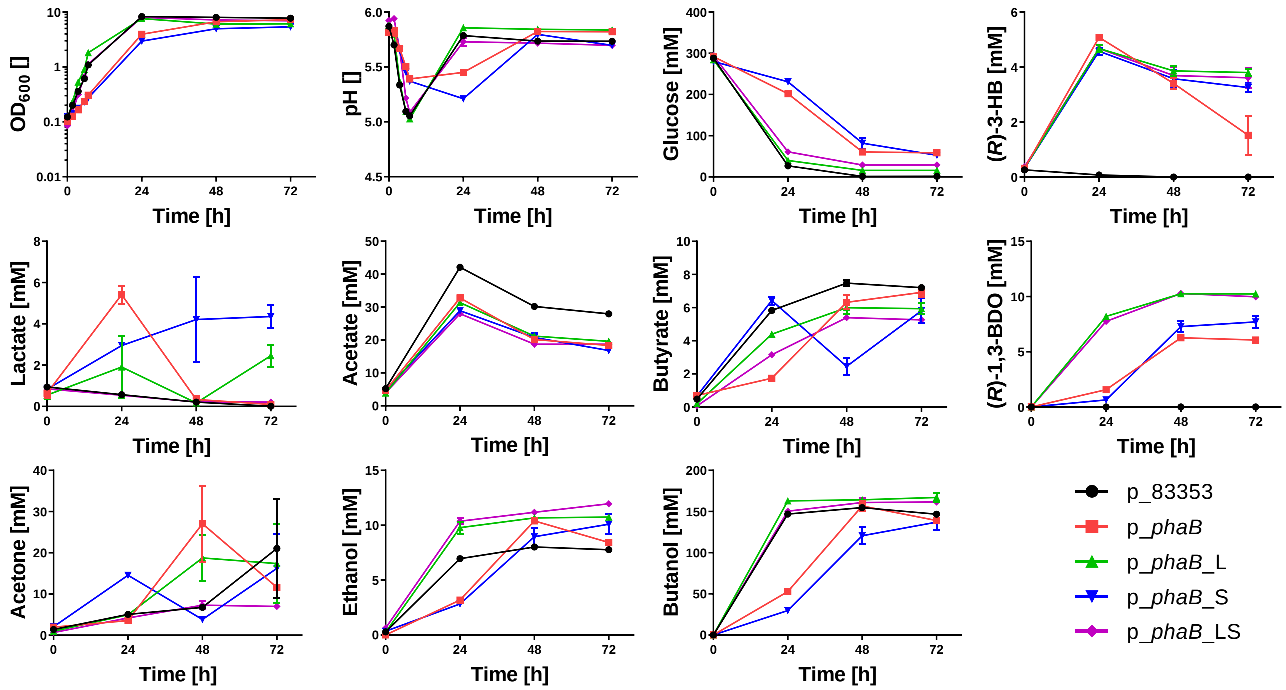


Figure S2. Comparison of product spectrum of *Cupriavidus necator* wild type PhaB and the derivatives with point mutations and negative control without *phaB* (p_83353) expressed in *C. saccharoperbutylacetonicum*. For each product p_82251 in black filled circles, p_*phaB* in red filled squares, p_*phaB*_L in green upwards pointing filled triangles, p_*phaB*_S in blue downwards pointing filled triangles, p_*phaB*_LS in purple filled diamonds. Error-bars represent SEM, n= 3.

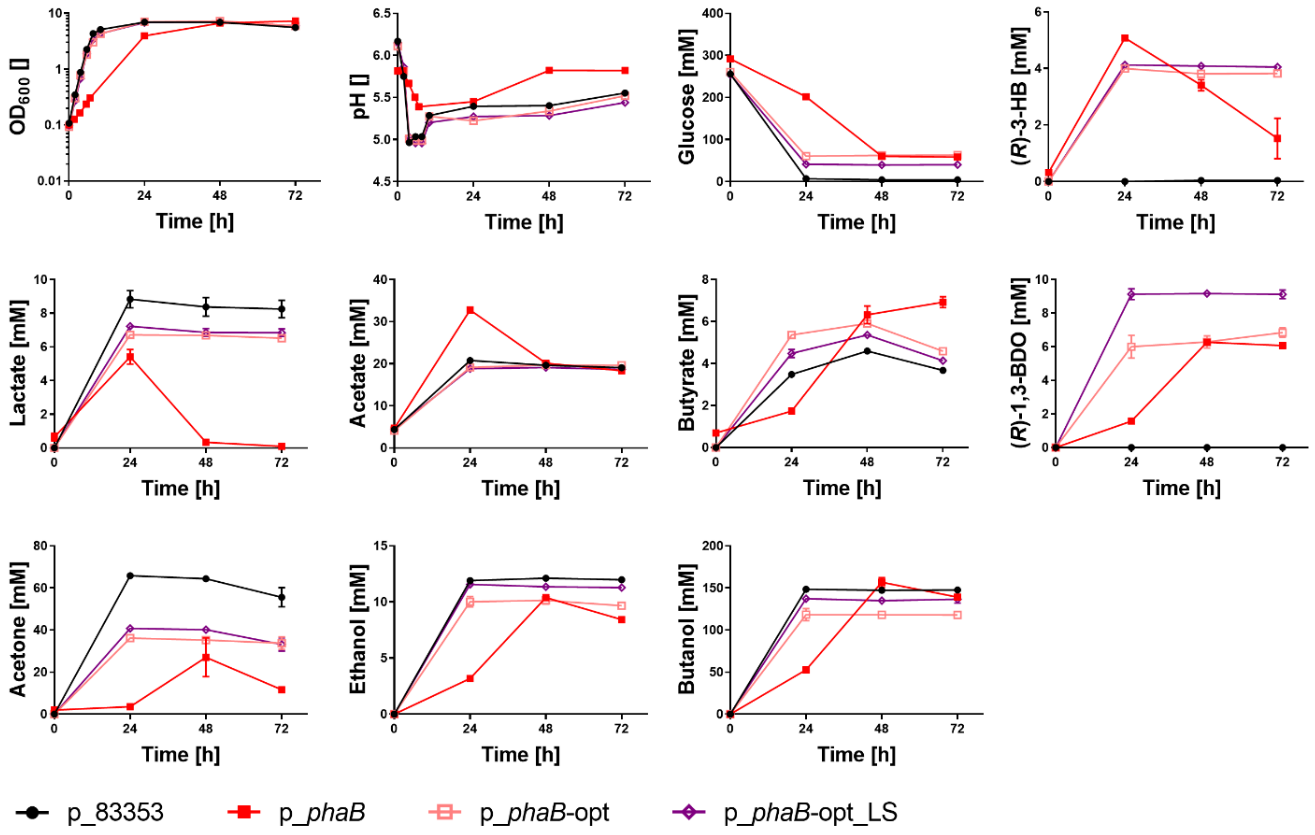


Figure S3. Comparison of product spectrum of *Cupriavidus necator* PhaB, the *C. saccharoperbutylacetonicum* codon optimised version and its derivative with point mutations expressed in *C. saccharoperbutylacetonicum*. For each product: p_83353 in black filled circles, p_*phaB* in red filled squares, p_*phaB*-opt in light red empty squares, p_*phaB*-opt_LS in purple empty diamonds. Error-bars represent SEM, n= 3 (apart from time point 0 where n= 1).

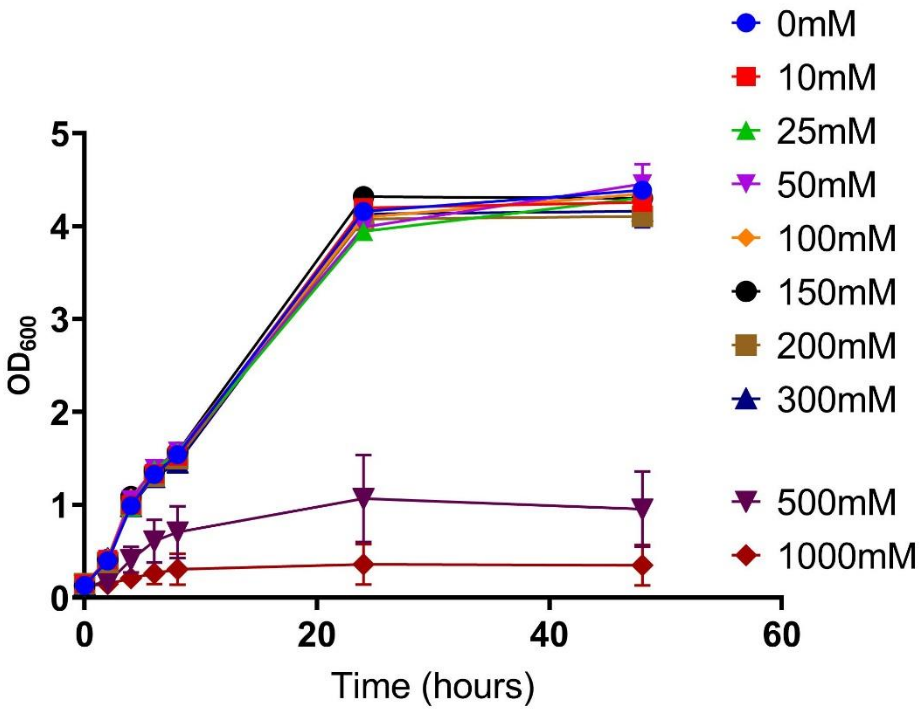


Figure S4. Growth of *C. saccharoperbutylacetonicum* on varying concentrations of *meso*-1,3-BDO. Growth curves were done as described in Materials and Methods, but stated concentrations of *meso*-1,3-BDO (Sigma-Aldrich, Dorset, UK) was added to the main cultures. Growth is inhibited by 500 mM *meso*-1,3-BDO corresponding to 45.06 g/l but is not inhibited by 300 mM corresponding to 27.04 g/l.

```
AA         R  E  R  W  L  E Q>L Q  K  A  L  G  F  D  F  V  A  S  E  G

phaB      AGAGAAAGATGGCTTGAACAGCAAAAAGCTTTAGGTTTTGACTTTGTAGCAAGTGAAGGA 180
phaB_L    AGAGAAAGATGGCTTGAACTGCAAAAAGCTTTAGGTTTTGACTTTGTAGCAAGTGAAGGA 180

phaB-opt  AGAGAAAGATGGTTAGAACAACAAAAGGCATTAGGATTTGATTTTGTTGCTTCAGAAGGC 180
```

Figure S5. Context of point mutation Q47L (in yellow) in a Clustal Omega alignment of wild type *C. necator phaB* (phaB), the point mutated (Q47L) gene (*phaB*_L) and the codon optimised version (*phaB*-opt). Codon-triplets up- and down-stream of the mutation are unchanged in all three versions. This could suggest the observed growth inhibitions of the native PhaB bearing strains (Figure S3) to be due to translational deficiencies due to codon co-occurrence similar to the suggestion by Corredu *et al*. 2019. Frequency of codon GAA to code for glutamate 82 %; frequency of CAG, CAA to code for glutamine is 11 % and 89 %, respectively; frequency of CTG to code for lysine is 2 %; frequency of CAA to code for glutamine is 83 %.

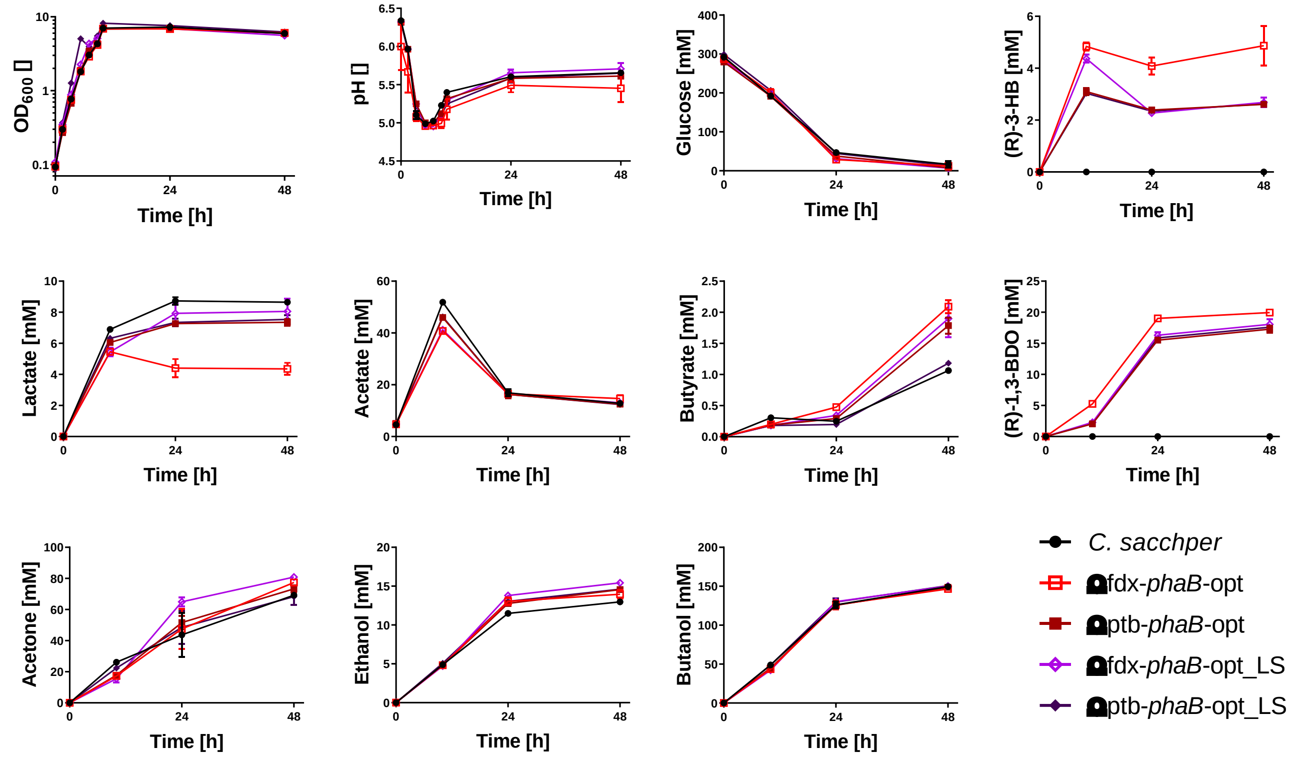


Figure S6. Comparison of product spectrum of codon optimised *phaB* with and without double point-mutation expressed from the chromosome with either weak spo_fdx promoter or strong cac_ptb promoter and wild type control. From left to right for each product *C. saccharoperbutylacetonicum* in black filled circles, Ωfdx-*phaB*-opt in red empty squares, Ωptb-*phaB*-opt in dark red filled squares, Ωfdx-*phaB*-opt_LS in purple empty diamonds, Ωptb-*phaB*-opt_LS in dark purple filled diamonds. Error-bars represent SEM, n= 3.

## References


Correddu, Danilo, José de Jesús Montaño López, S. Andreas Angermayr, Martin J. Middleditch, Leo S. Payne, and Ivanhoe K. H. Leung. 2019. “Effect of Consecutive Rare Codons on the Recombinant Production of Human Proteins in Escherichia Coli.” IUBMB Life, September, iub.2162. https://doi.org/10.1002/iub.2162.

Green, Edward, Dana Heldt, and Benjamin Bradley. "Method and microbes for the production of chiral compounds." U.S. Patent Application 16/082,505, filed April 18, 2019.

Heap, J T, O J Pennington, S T Cartman, and N P Minton. 2009. “A Modular System for Clostridium Shuttle Plasmids.” J Microbiol Methods 78 (1): 79–85. https://doi.org/DOI 10.1016/j.mimet.2009.05.004.

Heap, J T, M Ehsaan, C M Cooksley, Y K Ng, S T Cartman, K Winzer, and N P Minton. 2012. "Integration of DNA into Bacterial Chromosomes from Plasmids without a Counter-Selection Marker." Nucleic Acids Res 40 (8): e59. https://doi.org/10.1093/nar/gkr1321.

Willson, B. J. (2014) Genetic modification of *Clostridium acetobutylicum* for deconstruction of lignocellulose. [PhD thesis]. [Nottingham (UK)]: University of Nottingham
